# Is Single Cord Irradiation Going to Be a New Standard for T1a Glottic Carcinoma?

**DOI:** 10.3389/fonc.2020.01447

**Published:** 2020-08-27

**Authors:** Esengul Kocak Uzel, Metin Figen, Ömer Erol Uzel

**Affiliations:** ^1^Radiation Oncology Department, Sisli Hamidiye Etfal Education and Research Hospital, University of Health Science, Istanbul, Turkey; ^2^Department of Radiation Oncology, Istanbul University-Cerrahpasa Medical Faculty, Istanbul, Turkey

**Keywords:** glottic cancer, VHI (voice handicap index), single vocal cord irradiation, IMRT (intensity modulated radiation therapy), SBRT (stereotactic body radiation therapy)

## Abstract

**Purpose and Objective:** To evaluate the disease-free survival, overall survival, dosimetric, and voice handicap index (VHI) results of T1a glottic invasive squamous cell carcinoma (SCC) patients who underwent hypofractionated single vocal cord irradiation (HSVCI).

**Materials and Methods:** The data of 18 patients with stage T1a glottic SCC were collected prospectively and analyzed retrospectively between July 2016 and July 2019. Patients were immobilized using a custom-fitted thermoplastic face and shoulder mask in hyperextension position. The CT scan was performed with 1-mm-thick slices. A planned target volume (PTV) margin of 3 mm was given to clinical target volume (CTV) in all directions, and 13 organs at risk were identified. Patients were prescribed a total of 5760–5808 cGy in 15–16 fractions. Patients had daily cone-beam computed tomography (CBCT), and the treatment was carried out with the physician. VHI test was applied to patients before and at the end of radiotherapy (RT) and 1, 2, 3, 4, and 6 months after the completion of RT.

**Results:** Local control and overall survival rate is 100% for a median of 18 months (6–44 months) of follow-up. A patient was diagnosed with 2nd primary lung cancer and active treatment still continues. All patients completed the treatment within the scheduled time. Grade 1–2 dysphagia and dermatitis occurred in all patients, and no grade 3 and above side effects were observed. The mean values of VHI were 37.00, 39.83, 38.28, 17.17, 12.22, 8.56, and 6.06 at the beginning of RT, at the end of RT, and 1, 2, 3, 4, and 6 months after RT, respectively.

**Conclusion:** Compared to surgery and conventional laryngeal radiotherapy, HSVCI is an alternative treatment method for T1a glottic cancer by reducing the treatment time to 3 weeks, facilitating recurrence treatment, and providing effective sound quality without compromising local control. Considering that ~80% of recurrences in glottic cancer occur within the first 2 years, 100% local control in a median of 18 months is extremely successful, but long-term follow-up is essential to observe possible late side effects.

## Introduction

The larynx plays an essential role in daily and social life as it is responsible for voice production and coordination of respiration and swallowing; therefore, the treatment aim of laryngeal cancer is not just for better oncologic outcome but has to offer good functional quality. Glottic cancer accounts for 65–70% of all laryngeal cancers and majority of those patients are diagnosed in the early stages (1). Both laryngeal preservation surgery and radiotherapy are the standard treatment approach for early-stage glottic carcinoma with 5-year local control rates approaching 90% (2). T1a glottic cancer has an excellent 5-year local control rate approaching 95% (1). Both surgery and radiotherapy are well-established treatment modalities for T1 glottic cancer (2, 3). With the development of transoral laser surgery (TLS), TLS replaced open partial laryngectomy (OPL) (4) and claims of lower laryngectomy rates started to be reported with initial surgery over radiotherapy (5, 6). Although there is lack of randomized clinical trials, TLS supplanted classical conventional radiotherapy claiming to be less harmful to healthy tissues (7). On the contrary, radiotherapy has been found to enable slightly better voice quality compared to surgery in a randomized trial (8) and has a clear advantage over TLS in terms of VHI in a comparative study (9).

A total dose of 63–66 Gy with a fraction size of 2–2.25 Gy given one fraction per day, 5 days per week is widely accepted (10). Although conventional radiotherapy is given in different fractionation schemas, the entire glottic larynx is generally accepted standard treatment volume. Researchers from Erasmus University Medical Center developed “single cord radiotherapy,” which aims to target only the involved cord. Minimizing the irradiated volume resulted in lower dose received by non-involved laryngeal structures. Therefore, this resulted in diminished early complication rate and better voice quality may be achieved without compromising local control (11–15). After their publications, a new approach, “SBRT to the involved vocal cord,” for early glottic tumors has gained attention and has been investigated by other institutions (14, 16, 17). Mitigation of side effects from radiation exposure is very important for the group of patients who are prone to have tobacco-related vascular disease (15) and also likely to improve voice quality.

We adapted single vocal cord irradiation (SCVI) as standard treatment approach for T1a glottic cancers since 2016.

Here, we present the oncologic and voice handicap index (VHI) outcomes of 18 patients with early toxicity profile.

## Methods and Materials

Previously untreated 18 patients with stage T1a glottic laryngeal cancer [according to the American Joint Committee on Cancer (AJCC) tumor node metastasis (TNM) staging system, 8th edition] were treated with hypofractionated single cord RT between July 2016 and July 2019. Data were prospectively collected and retrospectively evaluated. All patients had a histologically proven squamous cell carcinoma of a single vocal cord (T1a). Patients with carcinoma *in situ* and dysplasia and patients with suspicious lesions elsewhere in the larynx were not included in the study. Before treatment, all patients were completely staged, using endoscopic examination and head and neck diagnostic CT scan. All patients provided informed consent before undergoing the treatment recommend by the radiation oncologist. Before submitting these data, an ethical committee approved the study (Hamidiye Sisli Etfal Teaching and Research Hospital ethical committee).

Patients were simulated and treated in supine position with hyperextension of chin and with arms on the side of the trunk, adequately positioned with a five-point fixated thermoplastic head and neck mask, in order to limit motion due to swallowing. Simulation was done with planning CT scan with a slice thickness of 1 mm for target volume delineation and organ at risk and patients were asked not to swallow during planning CT acquisition. Volume definition was made based on ICRU 50/62 (18, 19). CTV was the entire involved cord, and a 3- to 5-mm margin was added when visible tumor extends to the one end of vocal cord. PTV margin was 3 mm for all directions. The following structures were delineated as organs at risk (OARs): spinal cord, carotids (ipsilateral and contralateral), larynx, supraglottic larynx, arytenoids (ipsilateral and contralateral), thyroid cartilage, thyroid gland, constrictors, and cricopharyngeus muscle. Planning objective was to cover the entire PTV with at least 95% of prescribed dose and only 2% of PTV D2 was allowed >107% of the prescribed dose. Radiotherapy was delivered with the VMAT technique using a 6MV linear accelerator in three different radiotherapy centers.

Image-guided radiation treatment (IGRT), setup verification, and correction of the patients were performed by daily cone beam CT (CBCT). The thyroid cartilage was selected as the matching structure to set up correction for each fraction. Patients were asked not to swallow during CBCT acquisition and beam delivery. In between delivery of the beams, swallowing was allowed. All CBCT match was done by the physician for all fractions of the entire treatment.

Endpoints of the study were LC, VHI, overall survival (OS), and acute and late toxicity (based on Common Terminology Criteria for Adverse Events, Version 5). Acute toxicity is within 90 days and late toxicity is more than 90 days.

All patients are followed with physical and endoscopic examination in each visit; monthly for 6 months and bimonthly for 2 years every 3–4 months for 3 years. Yearly, low-dose CT of chest was also obtained for second primary lung cancer surveillance.

The present study also mentions voice quality problems in daily life. These problems were evaluated using a validated voice-specific questionnaire, the VHI (20). The VHI scores range from 0 to 120; a lower score corresponds to a good voice-related functional status. Total VHI scores of 10 or lower are considered normal. Voice quality assessment was done at baseline (before treatment), at the end of treatment, and at 1, 2, 3, 4, and 6 months after treatment.

## Results

**Figure 1:**
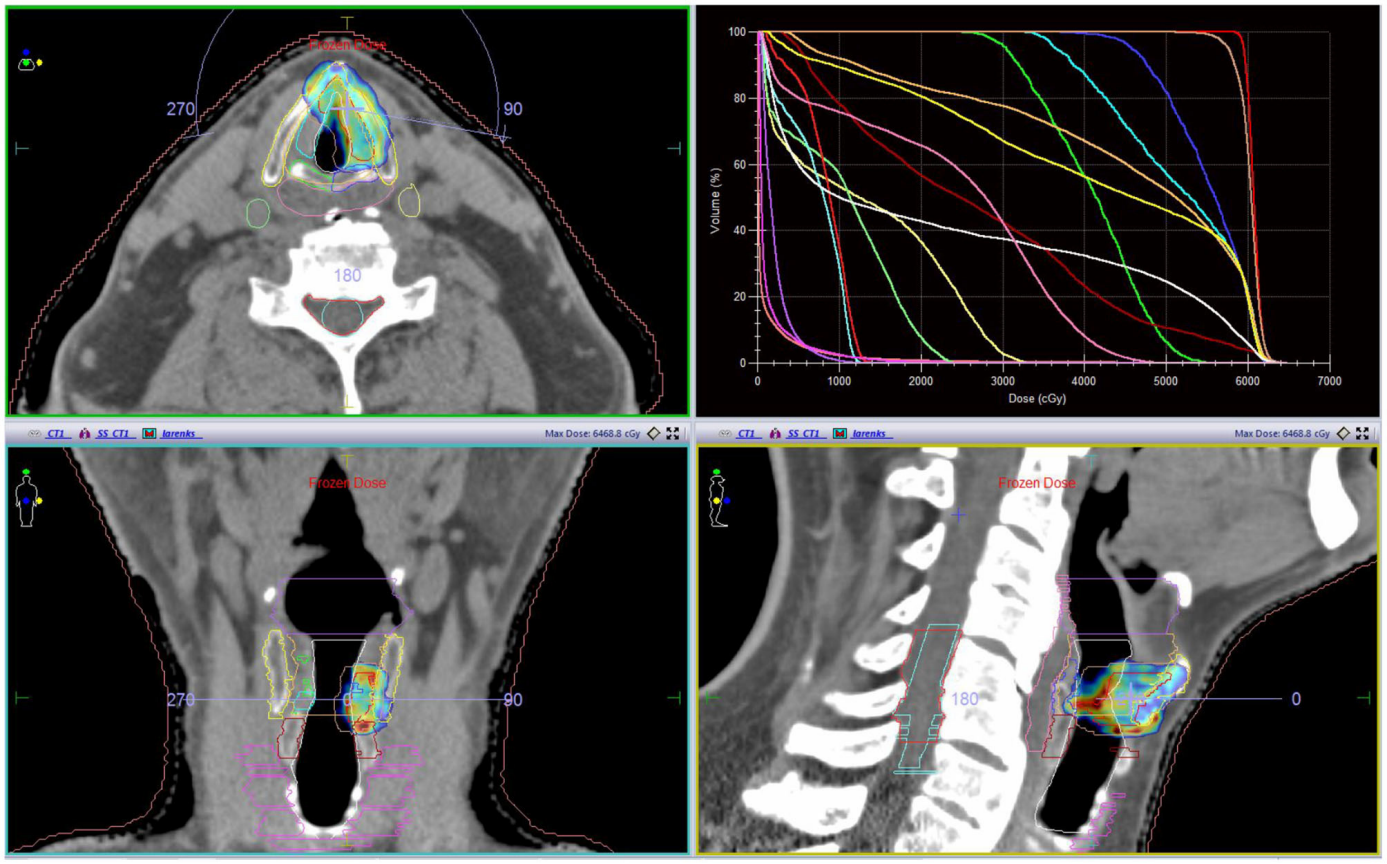
A representative case; tumor was located at the anterior third of vocal cord reaching but not involving anterior commissure; a 5-mm CTV margin was given in order to encompass possible microscopic disease (red line represents CTV). With OARs (Organ at risks) and DVH (dose volume histogram).

**Figure 2:**
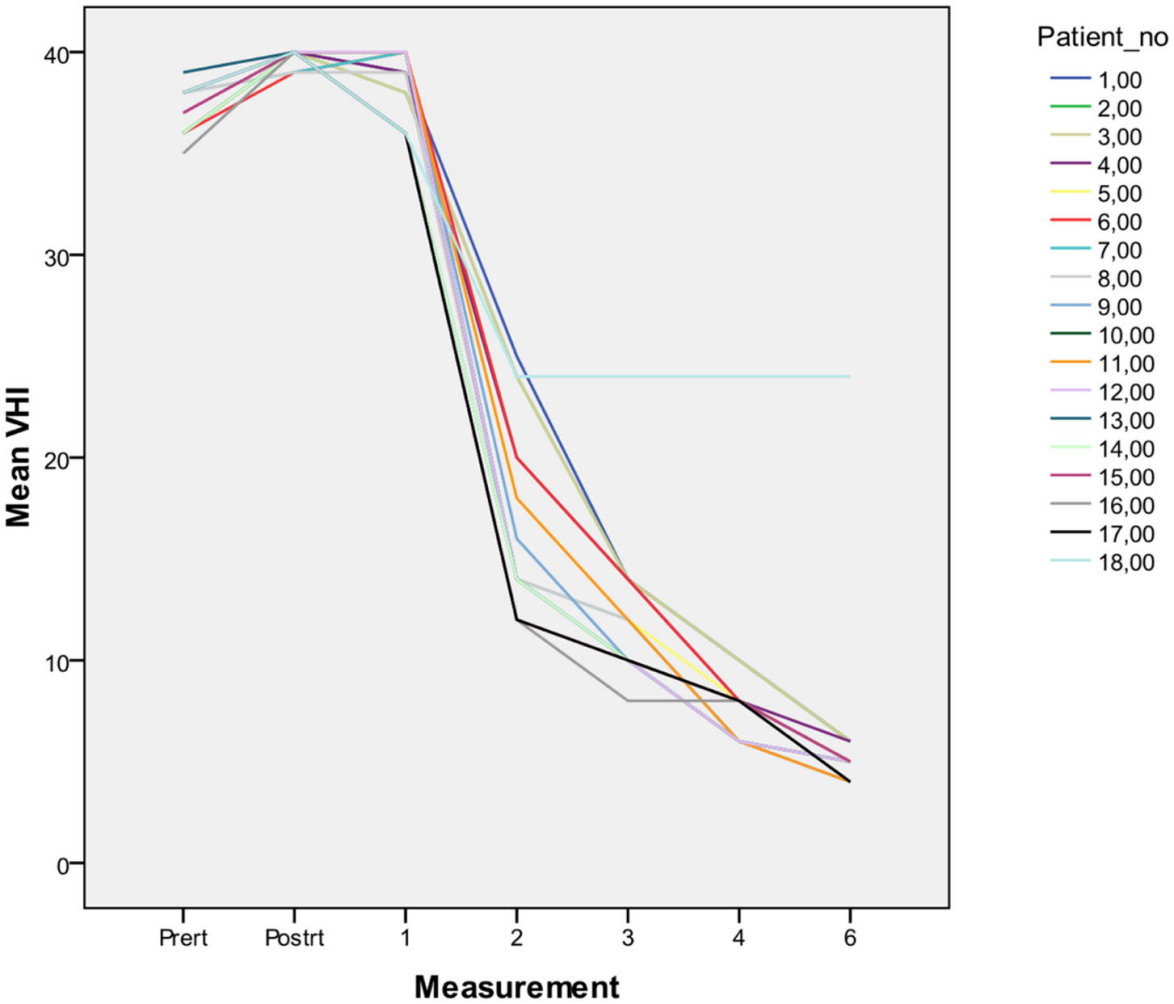
Voice quality assessment was done at baseline (before treatment), at the end of treatment, and at 1, 2, 3, 4, and 6 months after treatment. Results of all the cases are shown.

Among the entire group, 18 patients were male, and the median age was 70 years (range, 56–80 years). A total dose of 57.6–58.08 Gy was given in 15–16 fractions (median 58.08 Gy in 16 fr). The median overall treatment time (OTT) was 23 days (range, 22–26 days). All patients completed treatment as planned. An example of a plan is illustrated in Figure 1. Mean PTV volume, conformity index (CI), heterogeneity index (HI), and gradient index (GI) values are summarized in Table 1. After a median follow-up of 18 months (range, 6–44 months), the 2-year LC and OS rates were 100%; one patient was diagnosed to have second primary metastatic lung cancer 3 years after completion of radiotherapy; he is currently on systemic treatment. Of all patients who have completed the intended treatment schedule, no treatment interruptions and no grade 3 acute toxicity were reported. Acute dysphagia was observed in all patients, 12 grade 1(66%) and 6 grade 2 (33%). All patients had grade 1 acute dermatitis. So far, no serious late toxicity was observed. Mild ipsilateral arytenoid edema not requiring any treatment was observed in four patients. Voice quality assessment was done at baseline (before treatment), at the end of treatment, and at 1, 2, 3, 4, and 6 months after treatment. The results of VHI were 37.00, 39.83, 38.28, 17.17, 12.22, 8.56, and 6.06 respectively (Figure 2). Dosimetric results of OARs are summarized in Table 2.

**Table 1:** PTV volume, gradient index (GI), conformity index (CI), and heterogeneity index (HI).

	**Range**	**Mean ± SD**
PTV Volume_cc	4.17–9.84	6.59 ± 1.74
GI	4.40–7.90	5.16 ± 1.08
CI	1.16–1.60	1.33 ± 0.12
HI	1.09–1.18	1.11 ± 0.03

**Table 2:** Dosimetric results of OARs, IL (ipsilateral), and CL (contralateral).

**Organs**	**Dose range (cGy)**	**Mean ± SD (cGy)**
Spinal cord 0.1 cc	767–2,197	1387.89 ± 388.88
Spinal cord Max	2,314–3,208	2783.06 ± 322.42
IL_Carotid Max	2,314–3,208	2897.72 ± 308.80
IL_Carotid 0.1 cc	2,314–3,208	2746.72 ± 329.79
CL_Carotid Max	311–3,468	1576.17 ± 969.25
CL_Carotid 0.1 cc	240–3,110	1257.67 ± 878.90
Larynx (mean)	3,444–4,836	4050.39 ± 392.35
Supraglottic larynx (mean)	123–667	274.39 ± 168.54
CL_Arytenoid (mean)	1,101–4,080	2775.00 ± 862.73
Thyroid Cartilage (mean)	3,580–4,454	4091.67 ± 335.75
Thyroid gland (mean)	57–618	309.78 ± 167.36
CL_Vocal Cord (mean)	3,305–5,131	4319.78 ± 585.93
IL_Arytenoid (mean)	4,774–5,890	5554.78 ± 355.45
Constrictor (mean)	1,200–2,358	1677.44 ± 470.60
Constrictor V43Gy	0.69–21.44	5.57 ± 5.90
Constrictor V50Gy	0.01–16.10	2.67 ± 4.90
Cricopharyngus Mean	1,478–3,200	2552.06 ± 534.25
Cricopharyngus V43Gy	6.14–23.00	15.99 ± 5.71
Cricopharyngus V50Gy	3.44–14.00	9.69 ± 3.65

## Discussion

Single cord irradiation will be discussed with the following aspects: determination of CTV and PTV, and optimal dose fractionation regimen for maximum local control and minimal toxicity.

### Determination of CTV and PTV

Traditional radiotherapy fields for early glottic cancer is typically 5 by 5 cm lateral opposed fields targeting the entire glottic region with a generous margin (21). Vast majority of publications on radiotherapy for early glottic cancer report their results with this technique yielding local control rates of 85–95% (21, 22). IMRT has become the standard treatment for many head and neck sites for almost two decades; however, in the treatment of glottic cancer, adaptation of IMRT technique was rather late. First, IMRT use in early glottic tumors was aiming to reduce carotid doses while keeping the entire glottic region as a target (23). However, surgical treatment targets involved the cord or even the tumor itself with highly successful local control rates in experienced hands. The main reason for selecting the entire glottis as a target is actually the radiotherapy technique used in the past rather than field cancerization.

Consensus recommendations for delineating primary target volume for head and neck sites generally based on surgical pathological details and advised 0.5 cm from GTV are adequate for high-dose CTV (24). They also recommended an additional 0.5-cm margin for intermediate-dose CTV except for early glottic cancer (24). Probably after adaptation of new guidelines, majority of centers will use involved cord irradiation. Thus, encompassing the entire larynx in the treatment of early glottic cancer will no longer be valid.

In modern radiotherapy, stereotactic body radiotherapy (SBRT) is developed to treat small volume disease with high ablative doses with the aid of image guidance in various anatomical sites. Thus, we are now capable of targeting and treating the involved cord, which also allows us to spare normal tissues much more easily as the irradiated volume is smaller.

Target delineation is an essential part of treatment, and thin slice planning CT is mandatory. Researchers from Erasmus MC Cancer Institute tested the feasibility of single cord irradiation in a series of publications (11–13, 16, 25). Osman et al. (26) investigated vocal cord movement during breathing with 4D CT and concluded that breathing motion does not seem to be a limiting factor for SVCI. Then, the question arises how much PTV margin should be applied. Baron and colleagues looked at laryngeal movement relative to vertebral body with CT on rails and found that a 5-mm PTV margin would be adequate (27). Sher et al. treated their patients with Cyberknife; initially, they inserted fiducial markers in or in front of the thyroid cartilage and then on a tattooed spot of skin anterior to the thyroid cartilage to follow possible laryngeal movement, and they added 3-mm PTV expansion in all directions while using image guidance (16). Durmus et al. investigated laryngeal movement with intrafraction CBCT during treatment delivery choosing thyroid cartilage as a reference. They found that a 2-mm margin would be enough; at least 94.1% of the fractions delivered. Displacement to lateral direction was under 1 mm (28).

### Radiation Dose

For many years, 66 Gy in 33 fractions has been used as a standard until more hypofractionated regimen of 63 Gy in 28 fractions was introduced and proved to be better than 66 Gy/33 fractions in two randomized trials (29, 30). On the other hand, most centers in UK and Canada used more hypofractionated radiotherapy for glottic cancer with high local control rates (31, 32). Considering dose volume relationship on normal tissue complications, it is reasonable to increase the total dose if irradiated volume is decreased. The present study and a series by Al Mamgani et al. used almost the same dose fractionation schema 58 Gy in 16 fractions. It is calculated to be equivalent to 66 Gy in 33 fractions by BED formula when α/β ratio is 10.

Chung et al. used a standard fraction size of 2.25 Gy but treated their patients in 29 or 30 fractions reaching a total dose of 65.25 and 67.5 Gy (14). Sher et al. investigated three dose levels: 50 Gy in 15 fractions, 45 Gy in 10 fractions, and 42.5 Gy in 5 fractions; they concluded that 42.5 Gy in 5 fractions is feasible. A dose-escalating study conducted by Kang et al. should not be compared in terms of toxicity as they included the entire larynx at a certain dose level, but should be taken into account for local control. They used two dose levels for GTV: 59.5 Gy in 17 fractions and 55 Gy in 11 fractions; they observed one local recurrence in 13 patients (17). The abovementioned studies usually calculated dose EQD2 choosing an *a*/*b* ratio of 10 without time factor. BED3, BED10, and EQD2 for different dose levels are summarized in Table 3.

**Table 3:** BED3, BED10, and EQD2 values in studies evaluating SVCI.

**Dose/fraction (Ref.)**	**BED3**	**BED10**	**EQD2 (3)**	**EQD2 (10)**
58.08 Gy/16 (13)	128.4	79.17	77	66
59.5 Gy/17 (17)	128.92	80.33	77.35	67
50 Gy/15 (16)	105.57	66.67	62.3	54.86
45 Gy/10 (16)	112.5	65.25	67.5	54.37
42.5 Gy/5 (16)	162.97	78.62	98	65.5
63 Gy/28 (10)	110.27	77.21	66.16	64.32
65.25/29 (14)	114.22	79.94	68.53	66.62

Despite the fact that the majority dose levels mentioned above are considered to be equal to 66 Gy in 33 fractions for tumor control, time factor is neglected. However, overall treatment time plays an essential role in tumor control. To reduce the accelerated repopulation of tumor cells, shorter overall treatment time (OTT) with larger fraction sizes must be intended (33). Multiple series and metanalysis supported that OTT is an important prognostic factor in management of T1 glottic cancer (34). Voet et al. showed that tumor control rates decreased with increasing number of fractions and elapsed treatment time. OTT was the most significant factor for the locoregional control of T1 glottic cancer. Five-year local control rate decreased from 95% for 22–29 days to 79% for treatment time > 40 days (35). We obtained a maximal tumor control of 100% in a median 18-month period on a 22–23 day OTT period. When we assess the published data, the cutoff value for OTT is not conclusive yet. Recently, Shuryak et al. state that, optimizing fraction scheme to 18^*^3 Gy for head and neck tumors reduces late normal tissue complication probability and improves tumor control probability. From the point of early-stage tumors, an estimated tumor control probability from 82.9 to 87.9% and estimated reduction in late normal tissue complication from 13.1 to 1.4% can be obtained (36). It is expected that increasing the dose per fraction above 3Gy/fx is suboptimal because of unacceptably high late normal tissue complications. In the present series, so far 8 of 18 patients have been followed up more than 2 years and we have not witnessed any major or minor late complication. It might be due to smaller PTV volumes than traditionally irradiated.

### Local Control

Local control rate is 100% in the present series although follow-up time is limited. Al Mamgani et al. also reported 100% local control rate in 30 patients with a median follow-up of 30 months (13). Both studies used similar target volume and dose fractionation. A similar target volume description and technique but different fractionation is used by Chung et al. in their series of 34 patients with T1a glottic cancer; majority of patients were treated with 65.25 Gy in 29 fractions or 67.50 Gy in 30 fractions, and there was only 1 local recurrence in a median follow-up of 41.3 months (14). This patient was salvaged with partial laryngectomy. Sher and colleagues published a phase 1 fraction and dose escalation study for T1–2 glottic cancer (16). The following dose fractionation schedules were selected for study: level 0 50 Gy in 15 fractions (4 patients), level 1 45 Gy in 10 fractions (13 patients), and level 2 42.5 Gy in 5 fractions (12 patients). There were 2 local failures out of 4 in dose level 0 and 3 out of 13 in dose level 1. No local recurrence was observed in dose level 2. Three of five local recurrences were in patients with T2 tumors. One of the recurrences is considered to be a marginal miss. At a median follow up of 25.7 months, no recurrences were observed in dose level 2 (34). Kang et al. conducted a phase 1 clinical trial for SBRT in early glottic cancer with a different concept (17). They described two CTVs: one entire larynx and the second one with only gross tumor volume. They prescribed 47.6 Gy to larynx (PTV1) and 59.5 Gy to GTV (PTV2) in 17 fractions. For the second dose level, they prescribed 40.7 Gy and 55 Gy to PTV1 and PTV2, respectively. Treatment was delivered daily for dose level 1 and every other day or twice weekly for dose level 2. There was no local recurrence in seven patients in dose level 1. One local failure was observed 4 months after completion of radiotherapy in six patients treated with dose level 2. Results of previous and present studies are summarized in Table 4. Overall local control rates seem excellent for patients with T1 glottic cancer treated with single cord irradiation (17). Optimal dose and fractionation, however, will be determined in further studies.

**Table 4:** Summary of results of the investigated studies (^*^One of two recurrences occurred in a T2 tumor. **Two of three recurrences occurred in T2 tumors).

**References**	**No. of patients**	**Total dose (Gy)/fraction**	**T stage (Number of cases)**	**Local recurrence/Number of cases**	**Follow-up (months)**
Al Mamgani et al. (13)	30	58.06/16	T1a (30)	0/30	30
Sher et al. (16)	4	50/15	Tis (1), T1a (15), T1b (6), T2 (7)	2*/4	25.7
	13	45/10		3**/13	
	12	42.5/5		0/12	
Kang et al. (17)	7	59.7/17	T1a (4), T1b (2), T2 (1)	0/7	37
	6	55/11	T1a (5), T2 (1)	1/6	
Chung et al. (14)	34	65.25–67.5/29–30	T1a (34)	1/34	41.3
Present study*	18	58.06/16	T1a (18)	0/18	18

### Toxicity and Voice Quality

Dose to normal tissues is predictive of complication rates in radiotherapy. In the present series, we delineated 13 normal structures around the target and tried to keep dose to structures as low as possible. Al Mamgani also reported dose received by surrounding structures. Results of both studies were comparable. Ding et al. compared hemilarynx IMRT plans with SBRT; considerable reduction in contralateral arytenoid, ipsilateral and contralateral carotid, spinal cord, and thyroid gland doses was noted with SBRT plans with Cyberknife platform (37). In their clinical trial, only patients with high volume disease developed serious complications (16).

In the present series, we observed mild mucosal and skin toxicity in all patients; there was no grade III acute toxicity. Chung et al. reported 41% GII early mucosal toxicity and no late toxicity (14). Al Mamgani also reported no late toxicity; there was only one laryngeal edema that recovered with steroids (13).

In a phase I study conducted by Kang et al., laryngeal edema occurred in 3 out of 13 patients; 1 healed in 1 year, and the other 2 resolved in <3 months. Two of six patients developed GIII late laryngeal toxicity in 55 Gy in 11 fraction dose level. Trial was closed early because of high toxicity rate for early glottic cancer (17). High laryngeal edema rate is probably due to inclusion of the entire larynx as a part of target volume.

Another dose-escalating study was conducted by Sher et al. In their study, there were two dose-limiting toxicities (DLTs). One of them had a large-volume T2 tumor (PTV volume of 17 cm^3^), receiving 45 Gy in 10 fractions that actually appeared to be a true T4 tumor with cricoid cartilage involvement, and developed grade IV laryngeal edema 5 months after treatment requiring tracheostomy and gastrostomy; in 13 months, recurrence became obvious. A second DLT also occurred in a patient with a large T2 tumor (PTV volume of 21.3 cm^3^). One GII laryngeal edema was also observed in dose level 2 recovered with pentoxifylline and vitamin E. The latter two patients were exposed to heavy smoke (16). It appears that treatment volume plays an important role in the development of high-grade laryngeal edema. It can be concluded that it is feasible to irradiate small-volume disease with high-dose hypofractionated regimens. Nevertheless, one must be cautious as the follow-up times of these studies are not long enough to make absolute conclusions especially for late radiation effects.

An important aspect of treatment outcome for glottic laryngeal cancer is the voice quality. There are several methods to measure voice quality; although it is subjective, we used VHI to determine voice recovery after RT. VHI forms are filled and collected before, at the end, and at 1, 2, 3, 4, and 6 months after RT for each patient. Voice is recovered in all patients but one within 3 months after RT. Al Mamgani et al. compared VHI patients treated with SVCI and conventional whole larynx irradiation patients and concluded that less worsening at the end of treatment and better recovery starting from 6 weeks after RT are observed with SVCI (13). Both studies indicate that better voice recovery may be accomplished with SVCI; however, one must be cautious about long-term functional results as follow-up time is limited.

## Conclusion

Although we have a limited number of patients with short follow-up time, our result supports the use of SVCI for T1a glottic cancer. Daily image guidance is essential for high-precision delivery. Optimal dose and fractionation however are yet to be determined.

## Data Availability Statement

The raw data supporting the conclusions of this article will be made available by the authors, without undue reservation.

## Author Contributions

EK: data collection and writing. MF: data collection and helped to writing. ÖU: writing and data checking. All authors contributed to the article and approved the submitted version.

## Conflict of Interest

The authors declare that the research was conducted in the absence of any commercial or financial relationships that could be construed as a potential conflict of interest.
